# Total Wiring Length Minimization of *C. elegans* Neural Network: A Constrained Optimization Approach

**DOI:** 10.1371/journal.pone.0145029

**Published:** 2015-12-14

**Authors:** Andrey Gushchin, Ao Tang

**Affiliations:** 1 Center for Applied Mathematics, Cornell University, Ithaca, NY, United States of America; 2 School of Electrical and Computer Engineering, Cornell University, Ithaca, NY, United States of America; Georgia State University, UNITED STATES

## Abstract

Using the most recent data on the connectivity of the *C. elegans* neural network, we find optimal two-dimensional positions of interneurons that minimize the total wiring length provided that the positions of motor and sensory neurons are fixed. The rationale behind fixing motor and sensory neurons is the following: while positions of motor and sensory neurons can be influenced by the locations of muscles and sensory organs they are attached to, the main function of interneurons is to connect other neurons, and their placement could try to minimize the wiring length. Solutions for *l* ^1^, *l* ^2^ and squared *l* ^2^–norm were obtained. For the Euclidean norm *l* ^2^, the relative and absolute difference between the real and optimal total wiring lengths is minimal among these functions of distance. Additional network constraints were discussed such as assignment of different weights to electrical or chemical connections, fixation of “tail” interneurons, minimal interneural distance limitation, and others. These constraints were compared by their influence on the optimal positions of interneurons.

## Introduction

### Motivation

*Caenorhabditis elegans* is a transparent nematode which has been frequently used as a model organism and a subject of research for the past several decades. The nervous system as one of the main structures in the body of the worm has been thoroughly studied but still remains a topic of a special interest among the scientists. The nervous system of *C. elegans* is relatively well investigated in comparison with the other living organisms. Simplicity of the nervous system of *C. elegans* is one of the main factors that facilitates its study. The number of neurons in the hermaphrodite worm is only 302 with several thousand connections between them, and it was possible to extract information on the structure of the worm’s neural network, observe the neural cells and connections using light and electron microscopy. Among other things, the neural network of the *C. elegans*, including the number of neurons and their connectivity, do not significantly vary from animal to animal. In particular, number of neurons (302) in the hermaphrodite worm is consistent across the individuals [1, 2], and synapses (both chemical synapses and electric gap junctions) are stereotypical with more than 75% reproducibility [3]. This consistency makes it possible to obtain conclusions on the properties of the neural network of the whole species rather than of a particular organism.

Despite these properties of the worm that facilitate studying its nervous system, the data on connectivity of the neurons of *C. elegans* is still limited and inaccurate. Varshney et al. [3], using materials from [1] and new electron micrographs reported an updated set of data on electrical and chemical connections of the hermaphrodite worm. Although [3] was published in 2011, this updated connectivity dataset had been obtained before, and employed for example, in [4]. This refined dataset together with two-dimensional spatial locations of the *C. elegans* neurons in the animal’s body from [5–7] have been used in the present article. Similarly to previous works [4, 5, 8], by location of a neuron we imply the location of its cell body.

With this data on hand one can analyze whether the neural network is optimal in a certain sense provided that some constraints are satisfied. Various criteria of optimality can be applied and different constraints can be taken into account. For example, such metrics as the longest shortest path of the network, an average shortest path, total wiring length, number of connections—could both serve as the criteria of network optimality and as the constraints. In previous research [4, 5, 8] and in this paper as well, the criterion of optimality, that is the function to be minimized is the total wiring length—the sum of lengths of all connections in the neural network, and the constraints are the connections between the neurons. In other words, while keeping the neural connections fixed, we look for neurons positions such that the total wiring length is minimal, and compare this optimal placement with the real one. This choice of the network optimality criterion is based on the wiring economy principle in neuroscience [9–11]. According to this principle, wiring minimization has a significant influence on the brain organization, and neurons are arranged in an organism to minimize the wiring cost. Although it is not known what precisely defines the wiring cost, the cost of a connection between two neurons increases with the distance between them [4]. The total wiring length can be calculated in several ways depending on how the distance between two neurons is defined.

Furthermore, additional constraints on the cells placement can be applied. The main constraint that we consider in this article and that distinguishes it from existing works is the fixation of motor and sensory neurons at their real positions. These neurons interact with muscles and sensory organs of the worm, and their real positions can be influenced by this interaction, while real positions of interneurons could be explained by their interactions with other neurons. In this paper, by fixing motor and sensory neurons and altering positions of interneurons, we estimate to what extent locations of interneurons are determined by their interactions with other neurons and the wiring economy principle.

### Previous Research

In [5], Kaiser et al. analyzed two-dimensional positions of 277 *C. elegans* neurons. Using “component placement optimization” [12] they found a rearrangement of the neurons’ positions that minimizes the total wiring length. The wiring length of the optimal rearrangement was 48% smaller than the one of the real neural network. Rearrangement procedure is based on an iterative interchange of positions of two neurons, and thus, each neuron can be placed in one of the 277 predefined locations. Therefore, an optimization problem that does not incorporate such positional limitation, may provide a much larger reduction in the wiring length.

Ahn et al. [8] analyzed the total wiring length defined as a sum of the Euclidean lengths of the connections between neurons. Besides rearranging the neurons, they also employed the edge exchange shuffling method [13] in which two randomly chosen connections are replaced by the other two in such a way that the degree (number of connections from or to the cell) of each neuron remains unchanged on each step. Thus, the cell positions were fixed and the wiring was subject to change.

Chen et al. [4] solved for the optimal layout of the neurons using one-dimensional data on their positions in the worm’s body. Solutions for various distance functions have been found and compared to the real neuron placement. The closest to the real placement was the optimal layout obtained using the squared Euclidean distance (quadratic cost function).

### This Work Peculiarities

Depending on the function they perform in the body of the worm, neurons can be divided into three groups: motor neurons, sensory neurons and interneurons. The cells from the first group project their axons on muscles of the *C. elegans* and therefore control the movement of the worm. Sensory neurons help to process external information, for example touches or chemical signals. The main function of interneurons is to connect all other neurons together. Classification of the *C. elegans* neurons by the function they perform in the organism, is not complete because the functioning of some neurons remains unclear. Therefore, classifications from different sources have slight distinctions. For example, the number of interneurons varies from 76 to 86 depending on the source. As pointed out in [1], classification of neurons into three functional groups is not straightforward, because some of the neurons perform more than one function. Additionally, identification of sensory neurons is rather tentative due to lack of electrophysiological data.

In this paper, we fixed locations of motor and sensory neurons, and therefore, optimal positions of interneurons that minimize the total wiring length were obtained. The reason to anchor the neurons that implement motor and sensory functions is that these neurons are connected to muscles and sensory organs of the worm, respectively. Although the cell body of a neuron may not be important for communication in the neural network of *C. elegans*, the cell body positions of motor and sensory neurons are influenced by the locations of the muscles or sensory organs they interact with. In particular, they are generally located closer to the muscles and sensory organs as was demonstrated in [4], where locations of sensory organs and muscles were fixed, and the authors obtained the optimal positions of all neurons (motor, sensory and interneurons). The total wiring cost was expressed in [4] as a sum of an internal cost (to connect neurons to other neurons), and an external cost (to connect sensory and motor neurons to sensory organs and muscles). According to [4], the internal cost makes up to 91.7% of the total wiring cost. Therefore, assuming that the wiring economy principle underlies the network architecture, real locations of motor and sensory neurons can be explained by: (1) their interactions with muscles and sensory organs they are attached to; (2) their interactions with other neurons connected to them; (3) some other possible reasons such as, for instance, a tendency of some sensory neurons to be located in the nerve ring or tail ganglia; symmetry of the worm’s body; details of the neural development process. These reasons form constraints in the total wiring length minimization problem. While the first reason is relevant for sensory and motor neurons, the main function of interneurons is to connect other neurons, and not the interaction with sensory organs and muscles. Thus, if the network architecture is based on the wiring economy principle, one may expect that connectivity of interneurons with other neurons plays a dominant role in determining their locations, and one of the goals of this paper is to verify this hypothesis.

We should point out that synapses in *C. elegans* are formed *en passant* which may limit the validity of the analysis performed in this article. Majority of neurons, however, are nonbranching [4], and therefore the wiring length does not depend on number of synapses between any two neurons. To sum up,

- an updated wiring diagram from [3] and the spatial positions of neurons in a two-dimensional space from [5–7] were used,
- the total wiring length was used as a criterion of the network’s optimality,
- positions of motor and sensory neurons were fixed,
- positions of 86 interneurons were optimization variables,
- three distance functions have been employed.

In the next section we formulate and solve an optimization problem that minimizes the total wiring length of the worm’s neural network calculated using the squared Euclidean distance and *l* ^1^ and *l* ^2^–norms. We discuss the results and compare the optimal locations of interneurons and corresponding total wiring lengths obtained for these distance functions in the Results section. In the Discussion section we consider additional constraints on the network that could possibly explain the difference between the real and optimal positions of interneurons.

## Methods

In this paper we use the following data: the list of 279 nonpharyngeal neurons and their function (motor, sensory, or interneuron); chemical and electrical connections between these neurons; two-dimension physical locations of the neurons in the body of the worm. Chemical and electrical connections are represented by adjacency matrices *A* ^*ch*^ and *A* ^*el*^, respectively. For both matrices a matrix element *ij* is equal to one (if there is a connection from neuron *i* to neuron *j*) or zero (if this connections is absent). In other words,

$$\begin{array}{c} A_{ij}^{ch,el}=\{\begin{array}{cc} 1, & connection\;i\to j\;exists;\\ 0, & otherwise. \end{array} \end{array}$$ (1)

The chemical connections are directed, which means that a connection *i* → *j* from neuron *i* to neuron *j* and a connection *j* → *i* from neuron *j* to neuron *i* are two different connections. Hence, it is possible that there exists a chemical connection *i* → *j* from neuron *i* to neuron *j*, for example; however, connection *j* → *i* is absent. Thus, the adjacency matrix of the chemical connections *A* ^*ch*^ is not symmetric.

Electrical connections are bidirectional, and that is why the adjacency matrix of the electrical connections *A* ^*el*^ is symmetric. Two neurons *i* and *j* are electrically connected means that there are two directed electrical links: from *i* to *j*, and from *j* to *i*. In this paper by an electrical connection between neurons *i* and *j* we will assume the combination of these two links.

To combine information about chemical and electrical connections, we define a joint adjacency matrix: *A* = *A* ^*ch*^ + 0.5 * *A* ^*el*^. Here we multiplied the adjacency matrix of electrical connections by one half since a single electrical connection is represented by two ones in matrix *A* ^*el*^, and we assume that connections of chemical and electrical type contribute equally to the wiring length. Therefore, every connection regardless of its type has a unit weight in the estimation of the total wiring length. Under this assumption, elements of the adjacency matrix *A* can take one of these four values: 0, 0.5, 1, or 1.5. Other choices of weights are possible and will be discussed later in the text.

Therefore, the neural network of the *C. elegans* can be represented as a directed weighted graph, where the nodes of this graph are the neurons and two nodes *i* and *j* are connected by an edge *i* → *j* if and only if there exists a physical connection between corresponding neurons. The graph is directed, i.e. each edge has an associated direction, and weighted because two neurons can be connected by a chemical connection, by an electrical connection, by both types of connections, or are not connected at all.

Using the data on two-dimensional positions of neurons in the worm’s body, we can find the distances between each two neurons using various distance functions. In this paper, three functions for the distance have been employed corresponding to *l* ^1^, *l* ^2^ and squared *l* ^2^–norms. *l* ^1^—distance is defined as: $d_{1}\left(x,y\right)\;=\;\sum_{i=1}^{n}\left|x_{i}-y_{i}\right|$, the Euclidean distance *l* ^2^: $d_{2}\left(x,y\right)\;=\;\sqrt{\sum_{i=1}^{n}{\left(x_{i}-y_{i}\right)}^{2}}$, and the squared Euclidean distance: $d_{3}\left(x,y\right)\;=\;\sum_{i=1}^{n}{\left(x_{i}-y_{i}\right)}^{2}$, where *n* is a space dimension, *n* = 2 in our case. Squared Euclidean distance is formally not a distance function since it does not satisfy the triangle inequality property in the definition of the distance function. However, we will call it a distance function below for simplicity. From now on we will denote by *d*_*k*_(*i*, *j*) the distance between neurons *i* and *j*, where *k* = 1, 2 or 3, depending on the distance function. The relative distances between the neurons form distance matrices. Element $d_{ij}^{k}$ of a distance matrix *D* ^*k*^ is the distance between neurons *i* and *j*: $d_{ij}^{k}\;=\;d_{k}\left(i,j\right)$, *k* = 1, 2 or 3.

Now we define the elements of a weighted adjacency matrix *C* ^*k*^ as follows: $C_{ij}^{k}=A_{ij}*D_{ij}^{k}$. Thus, number $C_{ij}^{k}$ is positive only when there is a connection from a neuron *i* to a neuron *j*, and when it is positive, it is proportional to the distance between these two neurons.

The objective function of our optimization problem is the Total Wiring Length (TWL)—the sum of lengths of all connections in the neural network of the worm. In other words, TWL is the sum of all elements of the weighted adjacency matrix *C*. We assume that the connections in the network are fixed, i.e. matrix *A* remains unchanged. We also fix the two-dimensional positions of motor and sensory neurons. Therefore, the optimization variables of our problem are the positions of interneurons.

$$\begin{array}{c} \text{minimize}:\;\;\sum_{i,j=1}^{n}C_{ij}^{k},\\ \text{constraint}:\;\;motor,sensory\;neurons\;fixed. \end{array}$$ (2)

where *k* = 1 for *l* ^1^–norm, *k* = 2 for *l* ^2^–norm, and *k* = 3 for the squared Euclidean distance function.

A supplementary constraint obligates the interneurons to be located inside of the worm’s body, its addition to the optimization problem though does not change the solution.

## Results

### Optimization Results

**Fig 1:**
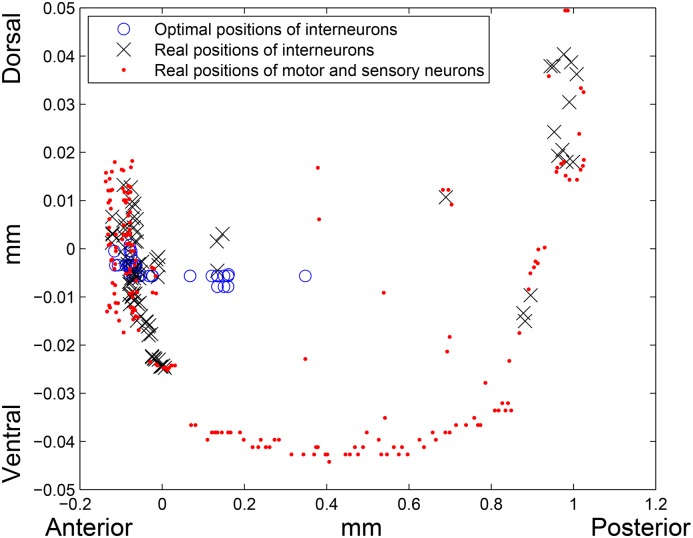
Real and optimal placement in *l* ^1^–norm. Red dots are the real locations of motor and sensory neurons, blue circles and black crosses are the optimal and real positions of interneurons, respectively. Left side of the picture corresponds to the head (anterior) region, right side corresponds to the tail (posterior) region of the worm, and bottom and top of the picture correspond to the ventral and dorsal sides of the worm, respectively.

For *k* = 1, that is, when *l* ^1^–norm is applied, the total wiring length of the network with optimal layout is 455.9401 mm, while the total wiring length of the real network in *l* ^1^–norm is 703.1003 mm. Thus, the reduction is 35.15%. An average *l* ^1^–norm distance between an interneuron’s real and optimal locations is 0.2220 mm (19.10% of the worm’s length). Both real and optimal placements of the neurons are depicted on Fig 1. If we place the interneurons randomly inside of the worm’s body, the total wiring length calculated using *l* ^1^–norm, on average will be around 1013.7 mm. To obtain this value, we assigned the interneurons positions randomly inside of the worm 1000 times and calculated the wiring length for each random arrangement. The average of these 1000 wiring lengths is equal to 1013.7 mm. Thereby, the total wiring length corresponding to the real interneurons’ layout is 30.64% lower than the average total wiring length corresponding to the random interneurons layout.

**Fig 2:**
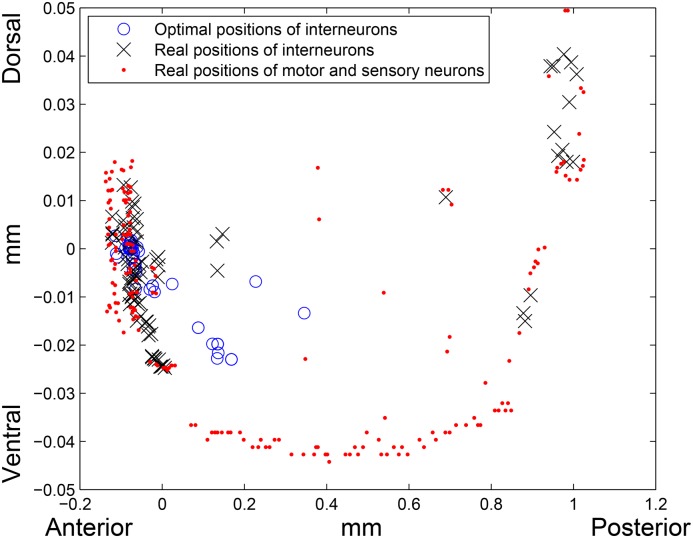
Real and optimal placement in *l* ^2^–norm. Red dots are the real locations of motor and sensory neurons, blue circles and black crosses are the optimal and real positions of interneurons, respectively. Left side of the picture corresponds to the head (anterior) region, right side corresponds to the tail (posterior) region of the worm, and bottom and top of the picture correspond to the ventral and dorsal sides of the worm, respectively.

If *k* = 2, that is, when *l* ^2^–norm (the Euclidean distance) is considered, the total wiring length of the network with the optimal layout is 430.2129 mm, while the total wiring length of the real network in *l* ^2^–norm is 662.8259 mm. Thus, the optimal interneurons locations reduce the total wiring length by 35.09%. Interestingly, that although the real wiring lengths in *l* ^1^ and *l* ^2^–norms differ by about 40 mm, the optimal positions of interneurons in both cases reduce the total wiring length by almost the same percent. An average *l* ^2^–norm distance between an interneuron’s real and optimal locations is 0.2104 mm (18.10% of the worm’s length). See optimal and real layouts on Fig 2. If the interneurons are placed randomly, on average the total wiring length will be equal to 954.85 mm. Therefore, the real total wiring length is 30.58% lower than the one estimated for an average random placement of the interneurons.

When the squared Euclidean distance is applied (*k* = 3), all distances are measured in squared millimeters. The total wiring length of the network with the optimal layout is 180.0027 mm^2^, while the total wiring length of the real network calculated using the squared Euclidean distance is 496.0584 mm^2^. Therefore, the reduction in the total wiring length for this distance function is 63.71%. The average squared Euclidean distance between the real and optimal locations of interneurons is 0.1041 mm^2^. It would not be reasonable, however, to directly compare this number with the corresponding numbers—0.2220 mm and 0.2104 mm—obtained for *l* ^1^ and *l* ^2^–norms respectively, since they have different units of measurement. Thus, this decrease of an average distance between the real and optimal interneurons positions for the squared Euclidean distance function is possibly explained by raising relatively small numbers (the Euclidean distances between the neurons) to the second power, rather than by the closeness of the real and optimal placements.

To check this hypothesis, we solved the TWL minimization problem using the third and the fourth powers of the Euclidean norm as the distance functions (as in a case with the squared Euclidean norm, these functions are not real distance functions, but we will call them so for simplicity). For the cubed Euclidean distance the real TWL is 450.7567 mm^3^, the optimal TWL is 93.6378 mm^3^, and optimal layout reduces the real TWL by 79.23%. The average cubed Euclidean distance between real and optimal positions of the interneurons is 0.0523 mm^3^. For the fourth power of the Euclidean norm we obtained the following results: 432.3243 mm^4^ is the real TWL, 58.6205 mm^4^ is the optimal TWL, i.e. 86.44% reduction. An average distance between real and optimal locations of the interneurons in this case is 0.0270 mm^4^. Therefore, as the exponent of the Euclidean distance increases, the difference between real and optimal TWLs increases as well, while the average difference between real and optimal positions of interneurons calculated by the corresponding distance function decreases. This decrease is caused by raising the numbers that are less than one into the second, third and fourth powers. Therefore, proximity between real and optimal layouts for each distance function should be measured by comparing the real and optimal TWLs, rather than by an average distance between real and optimal positions of interneurons without taking into account units of measurement of this distance.

**Fig 3:**
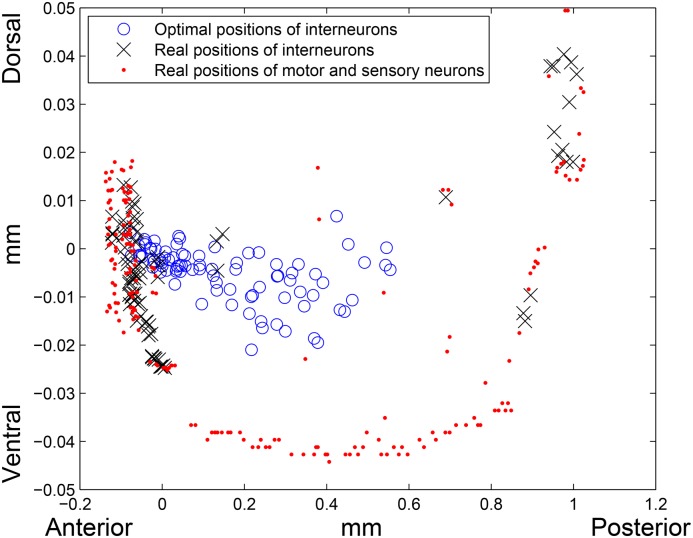
Real and optimal placement for the squared Euclidean distance function. Red dots are the real locations of motor and sensory neurons, blue circles and black crosses are the optimal and real positions of interneurons, respectively. Left side of the picture corresponds to the head (anterior) region, right side corresponds to the tail (posterior) region of the worm, and bottom and top of the picture correspond to the ventral and dorsal sides of the worm, respectively.

Thus, although the average distance between real and optimal locations of interneurons is smaller for the squared Euclidean distance function compared to both *l* ^1^ and *l* ^2^–norms, the optimal layout decreases the total wiring length for this distance function by 63.71%, which is about two times larger than the decrease for *l* ^1^ and *l* ^2^–norms. In addition, the average Euclidean distance between the real and optimal locations of interneurons calculated under the squared *l* ^2^–norm is $\sqrt{0.1041{\text{mm}}^{2}}\approx 0.3226\;\text{mm}$, that is larger than the corresponding value for the *l* ^2^–norm (0.2104 mm). The real and optimal positions of the neurons for the squared Euclidean distance function are plotted on Fig 3.

The average value of the total wiring length for the squared Euclidean distance function if the interneurons are placed randomly, is 609.2256 mm^2^. Therefore, the real total wiring length is only 18.58% lower than the one calculated for an average random allocation of the interneurons.

### Comparison of the Results

To sum up the results, *l* ^1^ and *l* ^2^–norms optimal layouts reduce the real total wiring length by nearly the same percent (35.15% and 35.09%, respectively), meanwhile the squared Euclidean norm optimal placement decreases the total wiring length by 63.71%. The absolute reductions of the total wiring lengths are: 247.1602 mm for *l* ^1^–norm, 232.6130 mm for *l* ^2^–norm, and 316.0557 mm^2^ for the squared Euclidean distance. Hence, for the Euclidean distance function, the difference (both absolute and relative) between the total wiring length corresponding to the real locations of interneurons and the total wiring length that corresponds to the optimal positions of interneurons, is the smallest one.

In [4], instead of motor and sensory neurons, the positions of muscles and sensory organs were fixed, and optimal one-dimensional locations of all 279 neurons were found. Although the authors did not compare the values of the real and optimal TWLs, they discovered that under the squared Euclidean distance function the optimal placement of neurons was the closest to the real layout. Interestingly, this conclusion is different from our results: if only locations of interneurons are optimized and two-dimensional positions of neurons are considered, the differences between the real and optimal TWLs, and real and optimal locations of interneurons are smaller for the Euclidean norm, and are the largest for the squared Euclidean distance.

**Fig 4:**
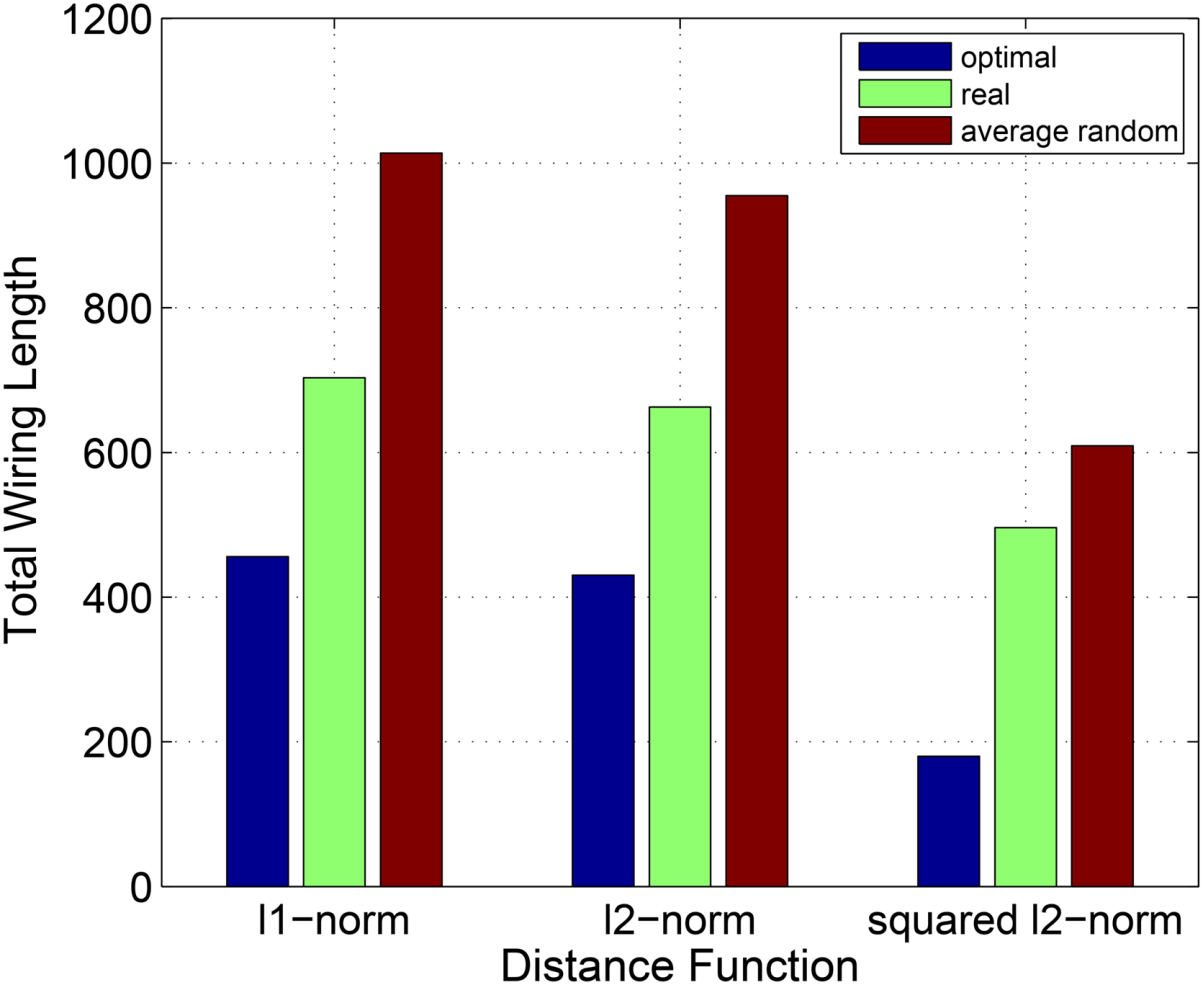
Total Wiring Lengths corresponding to optimal, real and average random placements of interneurons. The placements calculated using *l* ^1^, *l* ^2^ and the squared Euclidean distance functions. TWL is measured in mm for *l* ^1^ and *l* ^2^–norms and in mm^2^ for the squared Euclidean distance function. Blue bars are the optimal TWLs, green bars are the real TWLs, dark red bars are the average random TWLs. While for *l* ^1^ and *l* ^2^ distance functions the real TWL is closer to the optimal TWL than to an average random TWL, for the squared *l* ^2^ distance function the real TWL is closer to an average random than to the optimal TWL.

The real total wiring length is about 30% lower than the one corresponding to an average random placement of interneurons for both *l* ^1^ and *l* ^2^–norms, and only 18.58% lower for the squared Euclidean distance function. On Fig 4 optimal, real and random total wiring lengths are depicted for all three distance functions. For *l* ^1^ and *l* ^2^–norms, the real TWL is approximately in the middle between the values of optimal and average random TWLs.

## Discussion

### “Tail” Interneurons

**Fig 5:**
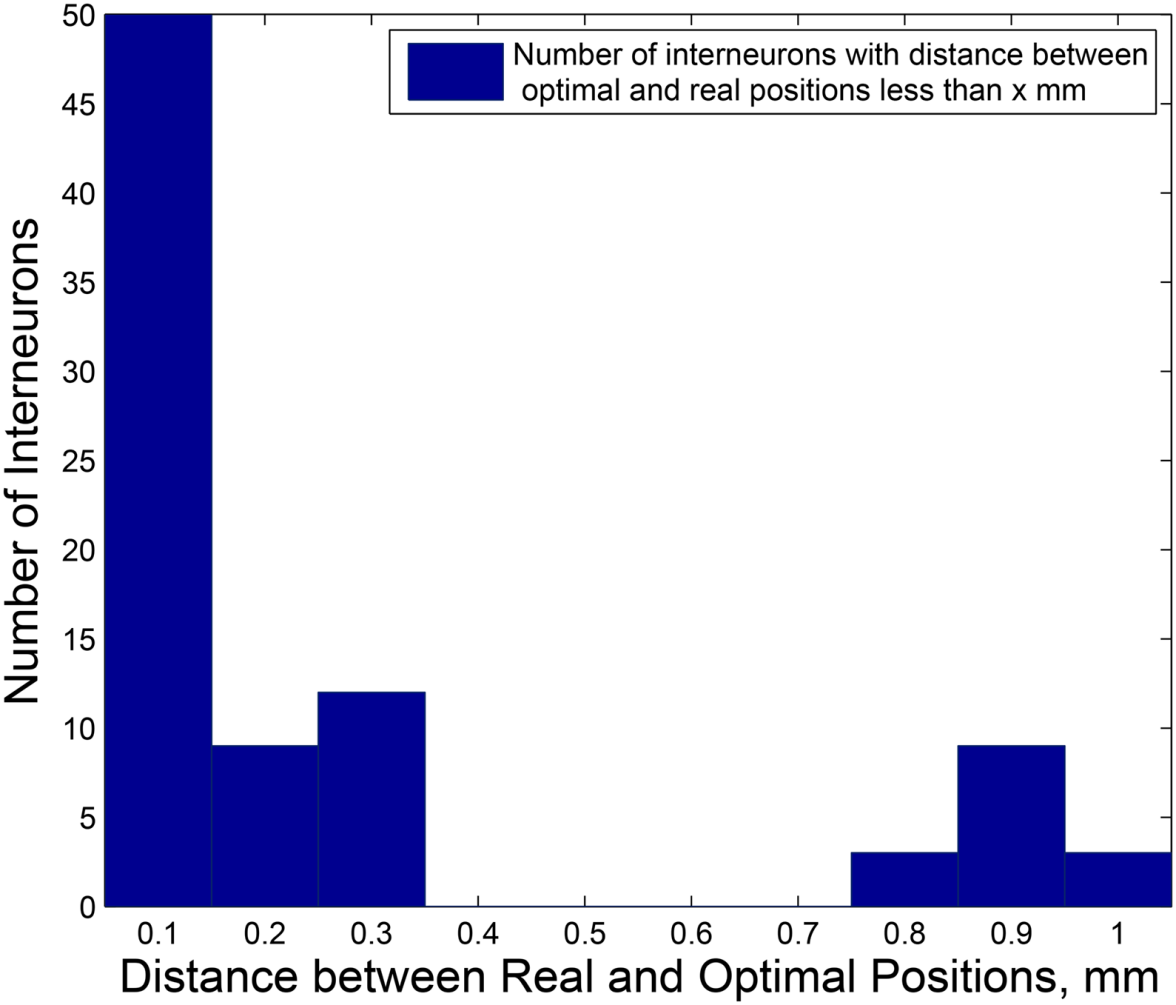
Distribution of interneurons by a distance between their real and optimal positions for the Euclidean distance function. Horizontal axis corresponds to the distance x between real and optimal positions of interneurons in mm, and vertical axis corresponds to the number of interneurons (out of 86) with the distance value x. For 50 interneurons the distance between real and optimal positions is less than 0.1 mm, for 15 tail interneurons the distance is greater than 0.7 mm, and there are no interneurons with the distance value between 0.3 mm and 0.7 mm.

If we compare the real and optimal positions of interneurons for all three distance functions, we can notice that there are several interneurons that are located in the tail region of the worm. Their optimal positions, however, are closer to the head region. From now on we will concentrate on the results for the Euclidean norm *l* ^2^. On Fig 5, distribution of interneurons by a distance between their real and optimal positions is plotted.

**Fig 6:**
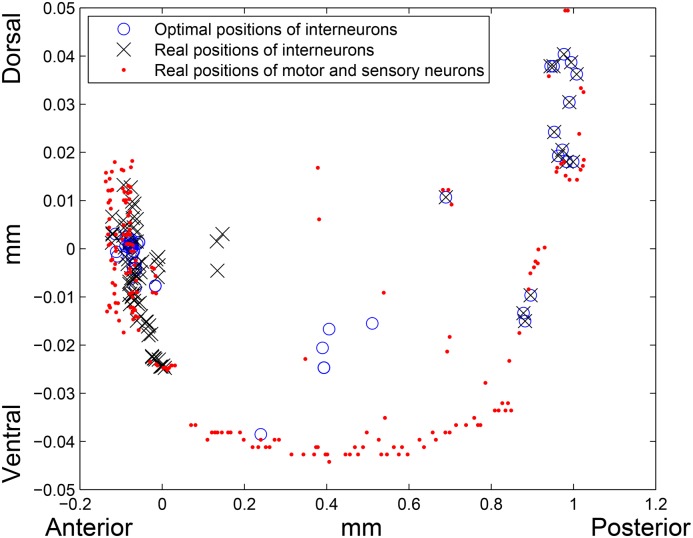
Real and optimal placement in *l* ^2^–norm (15 “tail” interneurons are fixed). Red dots are the real locations of motor and sensory neurons, blue circles and black crosses are the optimal and real positions of interneurons, respectively. Left side of the picture corresponds to the head (anterior) region, right side corresponds to the tail (posterior) region of the worm, and bottom and top of the picture correspond to the ventral and dorsal sides of the worm, respectively.

As we can observe, for the majority of interneurons (50 out of 86), the distance between the real and optimal locations is less than 0.1 mm. Moreover, an average distance between the real and optimal positions of interneurons in this group is only 0.0296 mm. At the same time there are 15 interneurons that contribute greatly to the difference between the real and optimal cell placements: SDQL, PVPR, PVPL, PVT, DVA, DVC, PVQR, PVQL, LUAL, LUAR, PVCL, PVCR, PVR, PVWL, PVWR. All of them are located in the tail region of the worm while their optimal positions are closer to the head region. To estimate an impact of these 15 neurons on the value of the total wiring length, we fixed them together with the motor and sensory neurons and solve the total wiring length minimization problems again. We obtained the following results: the total wiring length corresponding to the optimal positions of residual 71 interneurons is 582.4909 mm, which is only 12.12% smaller than the real total wiring length—662.8259 mm. The real cell placement together with the optimal placement obtained for 71 interneurons are depicted on Fig 6.

One of the possible reasons why these 15 interneurons are located in the tail region of the worm (instead of being closer to the head, what would minimize the wiring length), is that as was mentioned before, the classification of neurons by their function can be incomplete or inaccurate. It is possible that for some neurons their functionality is not fully identified. For example, some of the interneurons could also perform a motor or sensory function. It is possible that one or some of these tail interneurons are connected to muscles or sensory organs in the tail region, and it causes them to be displaced from the head. Indeed, in [14] it is mentioned that 3 of these 15 tail interneurons (SDQL, SDQR, DVA) additionally to their main function also implement a sensory function in the organism. In particular, SDQL and SDQR are oxygen-sensory neurons and DVA is a stretch sensitive sensory neuron. We predict that the remaining 12 tail interneurons can also be involved in performing sensory or motor functions in the worm.

### Minimal Interneural Distance

In the placements obtained by optimization problems some of the neurons are very close to each other, the distance between them is almost zero, meanwhile a neuron’s soma has a specific size, and the distance between two neurons cannot be less than a soma’s diameter. However, since we used two-dimensional data, i. e. projections of the neurons on a plane, the constraint on the minimal distance between neurons should not be applied. Indeed, two neurons with the positive distance between them in three dimensions, can even coincide when projected on a two-dimensional plane. Nevertheless, from a real two-dimensional positional data we can find the minimal distance between two neurons, 2.33 * 10^−4^ mm, and use that value as a minimal intercellular distance constraint in our optimization problem. With this constraint our optimization problem is not convex anymore, but the local optimal solution can still be found by taking the solution from previous section for *l* ^2^–norm as the initial guess. For the Euclidean distance function this additional constraint increases the optimal total wiring length from 430.2129 mm to 430.2610 mm, i.e. only by less than 0.01%. The additional constraint on the minimal distance between the neurons “pushes away” the neurons that are too close in the solution from the previous section, in such a way that this minimal distance constraint is satisfied.

### Using Number of Synapses and Gap Junctions as a Connection’s Weight

In previous sections we did not use information on the number of synapses in each connection. We assumed that all connections between neurons have equal weights. However, they differ in a number of chemical synapses (if it is a chemical connection) or electric synapses (if it is an electrical connection). To take this information into account, we redefine the chemical and electrical adjacency matrices *A* ^*ch*^ and *A* ^*el*^ in a following way:

$$\begin{array}{c} A_{ij}^{ch}=\{\begin{array}{cc} nsyn(ij), & connection\;i\to j\;exists;\\ 0, & otherwise, \end{array} \end{array}$$ (3)

where *nsyn*(*ij*) is the number of synapses for the chemical connection from a neuron *i* to a neuron *j*.

$$\begin{array}{c} A_{ij}^{el}=\{\begin{array}{cc} ngap(ij), & connection\;i\to j\;exists;\\ 0, & otherwise, \end{array} \end{array}$$ (4)

where *ngap*(*ij*) is the number of gap junctions for the electrical connection between neurons *i* and *j*.

**Fig 7:**
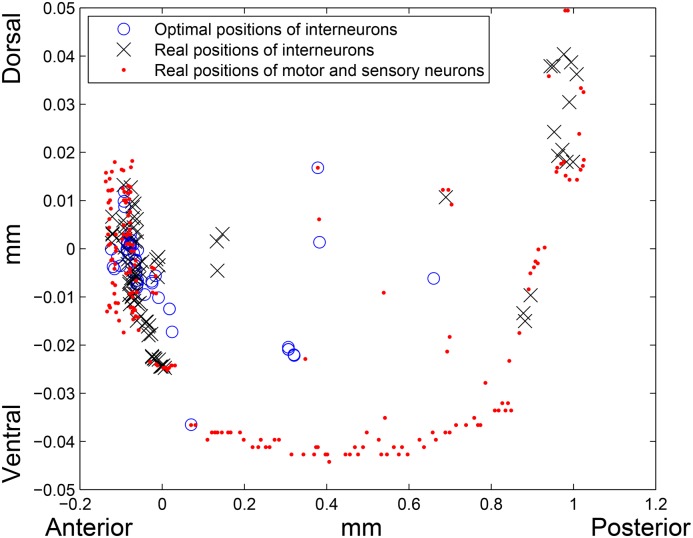
Real and optimal placement in *l* ^2^–norm when numbers of synapses and gap junctions are used as the connections weights. Red dots are the real locations of motor and sensory neurons, blue circles and black crosses are the optimal and real positions of interneurons, respectively. Left side of the picture corresponds to the head (anterior) region, right side corresponds to the tail (posterior) region of the worm, and bottom and top of the picture correspond to the ventral and dorsal sides of the worm, respectively.

Now, for example, a chemical connection *i* → *j* has a weight which is equal to the number of chemical synapses for this connection *i* → *j*, instead of having a unit weight. Using these newly defined adjacency matrices, we calculate all other matrices which are necessary for the TWL minimization problem in the same way as in section Results. The total wiring length of the real network calculated using these matrices is 1683.4 mm. Utilizing the Euclidean distance function, we solve for the interneurons positions that minimize the TWL. Real and optimal placements of the neurons are depicted on Fig 7.

The total wiring length that corresponds to the optimal layout of the neurons, is 1083.8 mm, and this is 35.62% less than the total wiring length of the real placement—1683.4 mm. The optimal positions of interneurons are slightly further on average from the real positions than the optimal positions of interneurons obtained in section Results for *l* ^2^–norm when number of synapses and gap junctions was not considered. Now, an average *l* ^2^–norm distance between an interneuron’s real and optimal locations is 0.2144 mm (was 0.2104 mm in section Results). An average distance between the optimal positions of interneurons obtained with and without considering number of chemical and electric synapses, is 0.0771 mm. Therefore, using number of synapses as the weights of neural connections only slightly changes the optimal positions of the interneurons. Interestingly, however, that on Fig 7 the optimal positions of “head” interneurons have a larger spread in the ventral-dorsal direction compared to the seemingly clustered around the head center optimal positions of the “head” interneurons on Figs 2 and 6.

### Assignment of Different Weights for Electrical and Chemical Connections

In this article we assumed equal weights for chemical and electrical connections. It might be possible, however, that for example, electrical connections have a bigger weight compared to the chemical connections, because two neurons with an electrical connection between them should be close to each other. This could be caused, for example, by necessity of fast interaction between the neurons, and electrical coupling can be faster than the chemical one (or vice versa), and the neurons should be located close to each other to minimize propagation delay. To figure out how changing the connections’ weights influences the optimal solutions, we solved two TWL minimization problem using the Euclidean distance function and assigning a weight of two to each electrical or chemical connection. In the first case, when all electrical connections have a doubled weight, the optimal interneurons positions reduce the real TWL by about 34.93%. When all chemical connections have a weight of two instead, the optimal layout reduces the TWL by about 35.41%. In the Results section, where both connection types had a unity weight, for the Euclidean norm we obtained a 35.09% reduction. Even though it looks like increasing the electrical connections weights makes the optimal TWL closer to the real TWL, the effect of changing the weights of connections on the difference between the real and optimal TWLs is relatively small. In extreme case, when the chemical connections are excluded and have a zero weight, the optimal allocation of interneurons diminishes the TWL by approximately 40.85%. Similarly, when all the electrical connections have a weight of zero, the optimal TWL is about 36.02% less than the real TWL.

Another possible choice is a binary weight assignment, when the weight *A*_*ij*_ is equal to one if there is a chemical connection from neuron *i* to neuron *j*, or an electric connection between these two neurons, and zero, otherwise. This weight assignment does not take into account information on number of connections and their type. For the binary weight assignment, the real TWL is closer to the optimal TWL compared to other choices of weight assignment: the optimal positions of interneurons reduce the wiring length by 33.08%.

In conclusion, most of the considered in this section constraints and supplements to the optimization problem formulated in section Results, have a relatively limited influence on the difference between the real and optimal total wiring lengths, and the optimal placement of interneurons reduces the TWL by about 35% in each case except for the binary weight assignment, where the TWL is reduced by 33.08%. However, fixing 15 tail interneurons diminishes this difference to slightly more than 10%.

One possible constraint that was not taken into consideration is the body symmetry. Some of the neurons, including interneurons, exist in pairs and often are located close to each other. For example, the distance between LUAL and LUAR, PVQL and PVQR, PVWL and PVWR, etc., is very small. This fact was not taken into account in our optimization problems.

## Conclusions

The goal of this article is to verify to what extent the neural layout of *C. elegans* can be predicted by the wiring economy principle. In contrast to the previous works on the optimality of *C. elegans* neural network, in our article we also took into account classification of neurons by the function they perform in the organism. While locations of motor and sensory neurons can depend on the locations of muscles and sensory organs they interact with, optimal locations of interneurons are mainly determined by their connections to other neurons and should minimize the wiring cost, if the wiring economy principle underlies the network’s architecture. Therefore, in this article we assumed that real positions of interneurons in *C. elegans* tend to minimize the total wiring length. To check how close the real TWL is to the minimal possible TWL, we obtained the optimal positions of interneurons that minimize the total wiring length. In addition, we calculated average TWL of the networks where interneurons are placed randomly. In our calculations, *l* ^1^, *l* ^2^ and squared *l* ^2^ distance functions have been employed. For each of these distance functions, the real TWL value was between the optimal and the average random ones. Nevertheless, the real TWLs calculated in *l* ^1^ and *l* ^2^–norms are closer to their corresponding optimal TWLs than to the average random TWLs, while the real TWL obtained for the squared Euclidean distance function is much closer to the average random TWL and not to the optimal total wiring length (Fig 4). For the Euclidean norm, optimal TWL (430.2129 mm) is 35.09% less than real TWL (662.8259 mm), while an average random TWL (954.85 mm) is 44.06% bigger than the real one. These results are different from the conclusions in [4], where the authors discovered that under the squared Euclidean norm the real positions of the neurons are closer to the optimal ones.

There are 15 interneurons in the worm’s body that are located in the tail region of the worm; their optimal positions, however, are closer to the head. Their “non optimal” real locations could be explained by additional not known at the moment function (sensory or motor) they might implement in the body. If positions of these 15 interneurons are fixed together with the positions of motor and sensory neurons, the optimal TWL is only 12.12% smaller than the real one. As mentioned in [1], classification of neurons is inaccurate, and it is especially difficult to determine whether a neuron is involved in performing a sensory function. In fact, 3 of the 15 tail interneurons are known to additionally carry out a sensory function. We therefore predict that the remaining 12 interneurons could also participate in sensory or motor functioning of the worm.

We also demonstrated that taking into account the actual number of synapses between connected neurons, or assigning different weights to chemical or electrical synapses does not have a significant impact on the optimization results. The first point can be explained by a fairly even distribution of synapses among the neural connections. The second fact could be caused by a similarity between the chemical and electrical connectivities and corresponding adjacency matrices.

Although in the real neural network of *C. elegans* the total wiring length minimization has indeed a large influence on the placement of interneurons, there is, however, a certain discrepancy between the real and optimal positions of interneurons. The possible reasons of this difference are the following: incomplete connectivity information, imprecise functional classification of the neurons, *en passant* formation of synapses, inaccurate wiring cost function, or non optimality of the real neural network in terms of the total wiring length. In this article we mainly explored two of these issues: the wiring cost function and classification of the neurons. To resolve the first issue, we determined the cost function (that is based on the Euclidean distance), for which the real placement is the closest to the optimal layout. For the second problem we listed 15 interneurons that could be misclassified.
